# Stability Profiles and Therapeutic Effect of Cu/Zn Superoxide Dismutase Chemically Coupled to *O*-Quaternary Chitosan Derivatives against Dextran Sodium Sulfate-Induced Colitis

**DOI:** 10.3390/ijms18061121

**Published:** 2017-05-24

**Authors:** Nan Zhao, Zhaolong Feng, Meng Shao, Jichao Cao, Fengshan Wang, Chunhui Liu

**Affiliations:** 1 Key Laboratory of Chemical Biology (Ministry of Education), Institute of Biochemical and Biotechnological Drugs, School of Pharmaceutical Sciences, Shandong University, Jinan 250012, China; xianggu8866@sina.com (N.Z.); zhaolong923688019@163.com (Z.F.); shaomeng0611@163.com (M.S.); caojichao@sdu.edu.cn (J.C.); fswang@sdu.edu.cn (F.W.); 2 National Glycoengineering Research Center, Shandong University, Jinan 250012, China

**Keywords:** superoxide dismutase, *O*-quaternary chitosan derivatives (*O*-HTCC), conjugate, stability, dextran sodium (DSS)-induced colitis, macrophage

## Abstract

Superoxide dismutase (SOD) has attracted considerable attention on treatment of reactive oxygen species (ROS)-related disorders. We previously conjugated Cu/Zn SOD to *O*-quaternary chitosan derivatives (*O*-HTCC) to yield a polymer–enzyme conjugate *O*-HTCC-SOD that demonstrated superior therapeutic effect to native SOD. The present study demonstrated that *O*-HTCC-SOD had wider pH activity range, better thermal stability, excellent long-term stability for storage, as well as unique reinstatement of activity exposure to proteolytic degradation that was helpful for longer half-life in vivo. *O*-HTCC-SOD exerted significant anti-inflammatory effects on lipopolysaccharides (LPS)-stimulated mouse peritoneal macrophages by down-regulating production of pro-inflammatory cytokines and intracellular ROS. *O*-HTCC-SOD significantly attenuated dextran sodium (DSS)-induced colitis in mice as observed by the colitis severity, neutrophil infiltration and histopathological damage, whereas native SOD failed to do so. In conclusion, conjugation of *O*-HTCC conferred SOD with better stability and enhanced therapeutic potential, offering a promising option in treatment of inflammatory bowel disease.

## 1. Introduction

Reactive oxygen species (ROS) is a collective term of oxygen-derived species, mainly including superoxide anion radical (O_2_^•−^), hydroxyl radicals (HO^•^) and hydrogen peroxide (H_2_O_2_). Under physiological conditions, intracellular ROS at low levels act as signaling molecules to support cell proliferation and survival pathways. However, excessive ROS over the cellular antioxidant defense system can cause deleterious oxidative stress, which has been implicated in diverse severe diseases [1,2,3,4,5]. Among of these diseases, inflammatory bowel disease (IBD) is a chronic, relapsing and remitting inflammatory disorder of the bowel and consists mainly of Crohn’s disease (CD) and ulcerative colitis (UC) [6]. Although the exact etiology of IBD remains uncertain, a positive correlation between the severity of IBD and the intestinal level of ROS has been reported [7].

Cells have evolved a series of anti-oxidative defense systems to counteract these highly dangerous and extremely reactive insults [8,9]. In the enzymatic defense systems, the most crucial enzyme to overcome the potential toxicity of ROS is superoxide dismutase (SOD). It catalyzes the dismutation of the most dangerous superoxide anion to hydrogen peroxide that is subsequently detoxified to oxygen and water by catalase or glutathione peroxidase [10]. Thus, SOD enzyme and other ROS scavengers have been found to be a potentially effective agent for colitis treatment [11,12,13]. However, the major drawback of systemic administration of SOD is its ultrashort elimination half-life (5–10 min) and poor permeability across the membranes into cells where the superoxide anion is produced [14]. 

Chemical modification of SOD is proven to be promising in controlling its disposition characteristics in the body thus improving its pharmacological activity. The pegylated SOD (PEG-SOD) had a significant longer circulation time, nevertheless did not yield a satisfactory therapeutic outcome in vivo due to its decreased permeability across the membranes [15]. The lecithinized Cu/Zn-SOD (PC-SOD) has received wide attention on potentially benefits in the prevention of experimental colitis [16]. In our previous studies, Cu/Zn superoxide dismutase (SOD) was successfully attached to a biodegradable chitosan derivative, *O*-(2-hydroxyl) propyl-3-trimethyl ammonium chitosan chloride (*O*-HTCC), to form a novel polymer-enzyme conjugate, *O*-HTCC-SOD. The conjugate has demonstrated its low cytotoxicity to cells, superior membrane permeability to native SOD, prolonged half-life and increased bioavailability in vivo, which consequently attenuated ROS-induced oxidative damage and induced tissue protection in mice. The *O*-HTCC-SOD conjugate might be a promising therapeutic agent for treatment of ROS-related diseases [17]. 

It has been confirmed that dextran sodium sulfate (DSS) is directly toxic to gut epithelial cells of the basal crypts and affects the integrity of the mucosal barrier [18]. DSS-induced murine experimental models have proved to be high resemblance of human UC in many pathological symptoms such as diarrhea, bloody feces, body weight loss and shortening of the colorectum. In this study, we firstly investigated the stability profiles of *O*-HTCC-SOD conjugate exposure to various stresses including temperature, pH, proteases, verified the effect of *O*-HTCC-SOD conjugate on the pro-inflammatory cytokines and the intracellular ROS levels in LPS-stimulated mouse peritoneal macrophages, and finally explored therapeutic effects of *O*-HTCC-SOD conjugate against DSS-induced acute colitis in mice. The data allowed us to evaluate whether the conjugate could serve as a new clinically therapeutic option for treating patients suffering from IBD.

## 2. Results and Discussion

### 2.1. Stability Profiles of O-Quaternary Chitosan (O-HTCC)-Cu/Zn Superoxide Dismutase (SOD)

Preparation of therapeutic proteins is often prone to instability during storage and use, and enhanced structural stability is a desirable attribute of modification technologies, but pegylation does not necessarily stabilize proteins to external stressors [19,20]. We have previously chemically conjugated Cu/Zn superoxide dismutase (SOD) to *O*-quaternary chitosan derivative to yield a novel polymer–enzyme conjugate, *O*-HTCC-SOD. Here, the enzymatic activity of *O*-HTCC-SOD was evaluated before and after exposure to various stresses.

**Figure 1:**
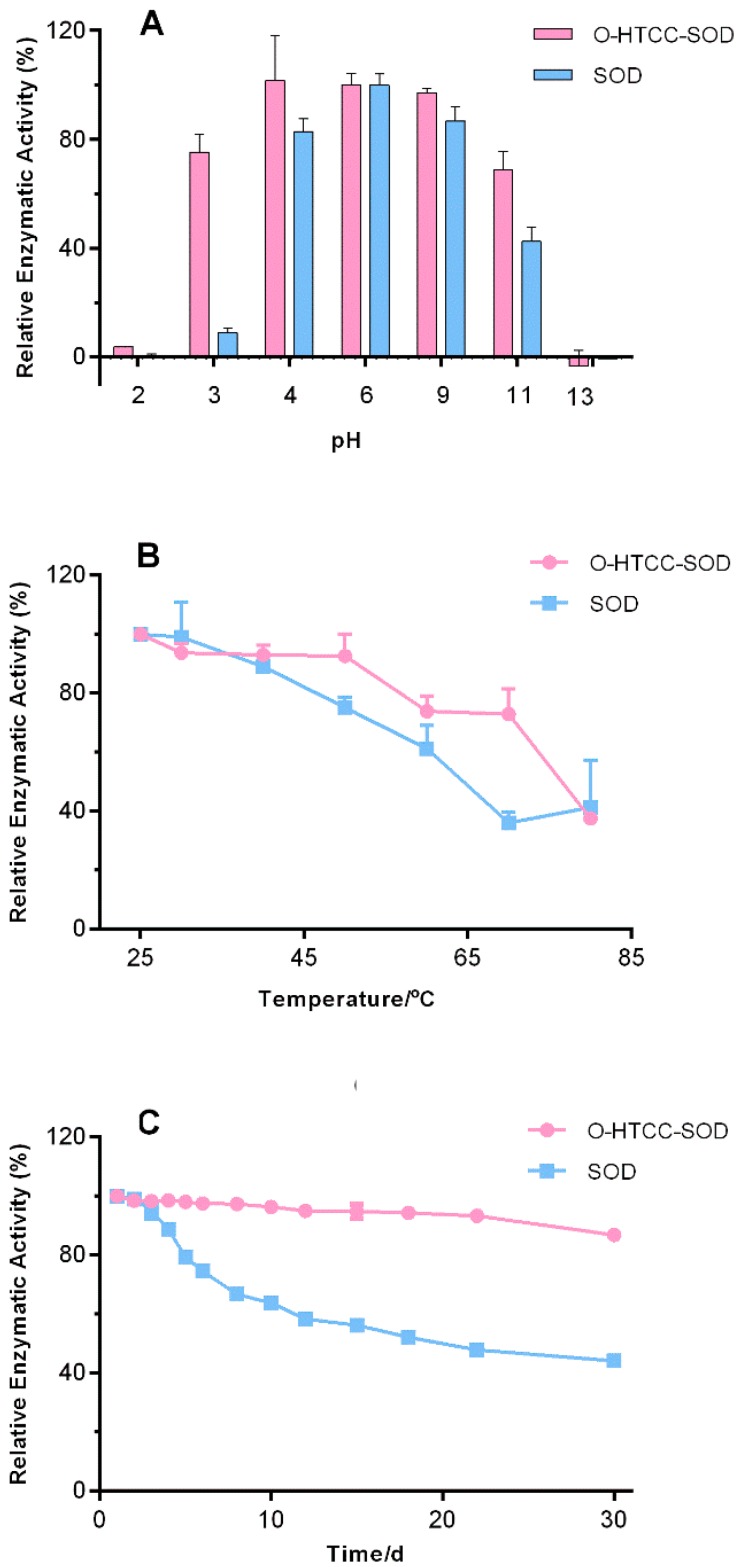
Stability profiles of native Cu/Zn superoxide dismutase (SOD) and O-HTCC-conjugated SOD (O-HTCC-SOD) exposure to: different pH (**A**); different temperature (**B**); and 30-day storage at room temperature (**C**). The relative enzymatic activity was expressed as percentage of the initial activity. Each experiment was carried out in triplicate using one batch of native SOD and *O*-HTCC-SOD. Data are presented as mean ± SD (*n* = 3). d = day.

Both unmodified and *O*-HTCC-conjugated SOD had no obvious loss of enzymatic activity under moderate pH (pH 4.0–9.0) conditions, as shown in Figure 1A. Exposure to extremely acidic condition of pH 3.0 reduced the enzymatic activity of *O*-HTCC-SOD by 25%, while >90% for native SOD. The residual enzymatic activity of the conjugate also was higher at extremely alkaline condition (pH 11) than native SOD. Thus, *O*-HTCC-SOD was more stable toward extreme acidic and alkaline conditions, suggesting modification of *O*-HTCC had a wider pH range of enzymatic activity. The thermal stability was shown in Figure 1B. There was no significant difference in enzymatic activity between native SOD and *O*-HTCC-SOD in the appropriate temperature range (25–40 °C). The native SOD rapidly decreased its activity from 90% to only 30% when the temperature was elevated from 40 to 70 °C. However, *O*-HTCC-SOD retained about 75% of its initial activity of SOD at 70 °C. Obviously, conjugation to *O*-HTCC significantly increased the thermal stability of the enzyme. Based on Figure 1C, there were sustained downward trend for the activity of native SOD and *O*-HTCC-SOD. The residual activity of SOD was only 44.1% after 30-day storage at room temperature, while that of the conjugate was up to 86.8%. The excellent long-term stability would be helpful to prolong shelf-life for SOD. These results demonstrated the importance of an *O*-HTCC polymer for the observed protective effect against various stressors. As our previous study [17], the conjugation led to random coil reduction and α-helix increase. The greater structural mobility from unordered structure to a highly ordered secondary structure might help to improve the stability of SOD.

**Figure 2:**
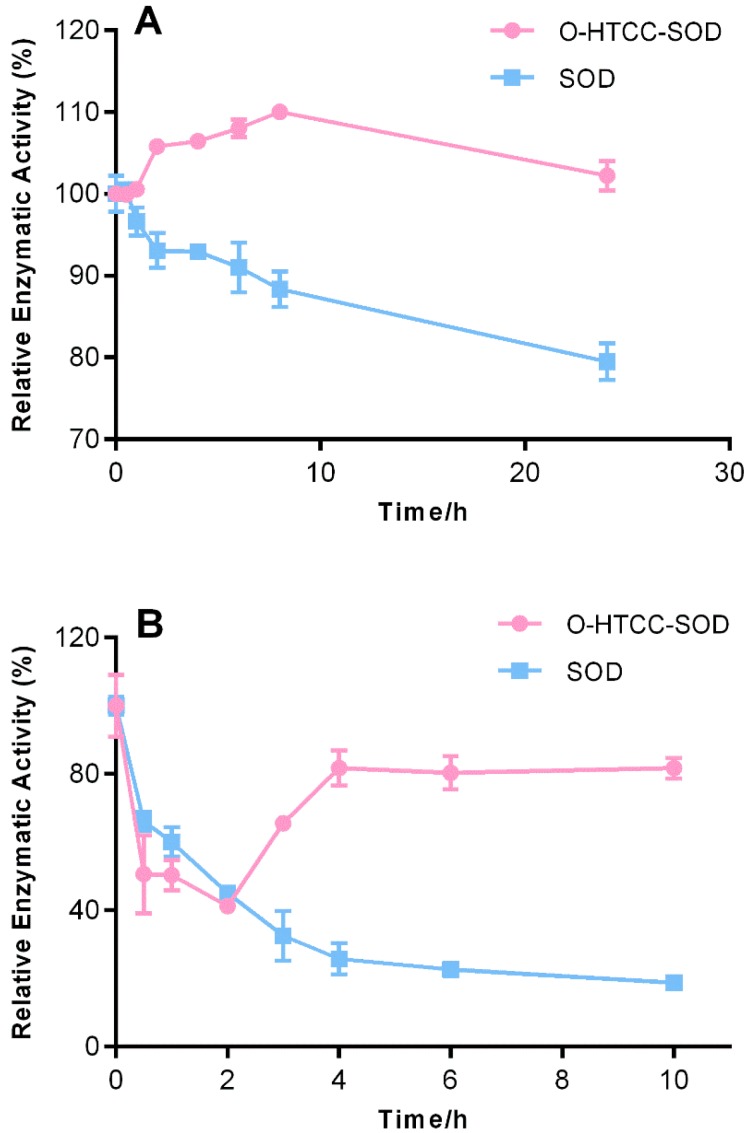
Time courses of relative activity of native SOD and *O*-HTCC-SOD incubated with: trypsin (**A**); and pepsin (**B**). The relative enzymatic activity was expressed as the percentage of the initial activity. Each experiment was carried out in triplicate using one batch of native SOD and *O*-HTCC-SOD. Data are presented as mean ± SD (*n* = 3).

It has been generally accepted that short half-lives and poor bioavailability of therapeutic proteins result from sensitivity to proteases and rapid renal clearance. Thus, their stability towards proteolytic hydrolysis was investigated. Treatment of trypsin reduced enzyme activity of native SOD by 20% within 24 h, but *O*-HTCC-SOD showed no obvious loss of SOD activity during the period of incubation with trypsin (Figure 2A), indicating *O*-HTCC-SOD had more resistant to trypsin degradation. Similarly, treatment of pepsin led to enzymatic activity decrease of native SOD by ~80% within 10 h. Interestingly, treatment with pepsin decreased the catalytic activity of *O*-HTCC-SOD to ~40% in the first 2 h, nevertheless, the enzymatic activity was restored to a maximum of 80% during additional incubation with pepsin and was maintained for at least 10 h (Figure 2B). The unique reinstatement of activity triggered by pepsin, regardless of the exact reason, was related to the gradual enzymatic degradation of *O*-HTCC coupled with SOD and helpful for the enzyme to prolong half-life and enhance the bioavailability. 

### 2.2. O-HTCC-SOD Reduced Pro-Inflammatory Cytokine Release in Lipopolysaccharides (LPS)-Stimulated Macrophages

**Figure 3:**
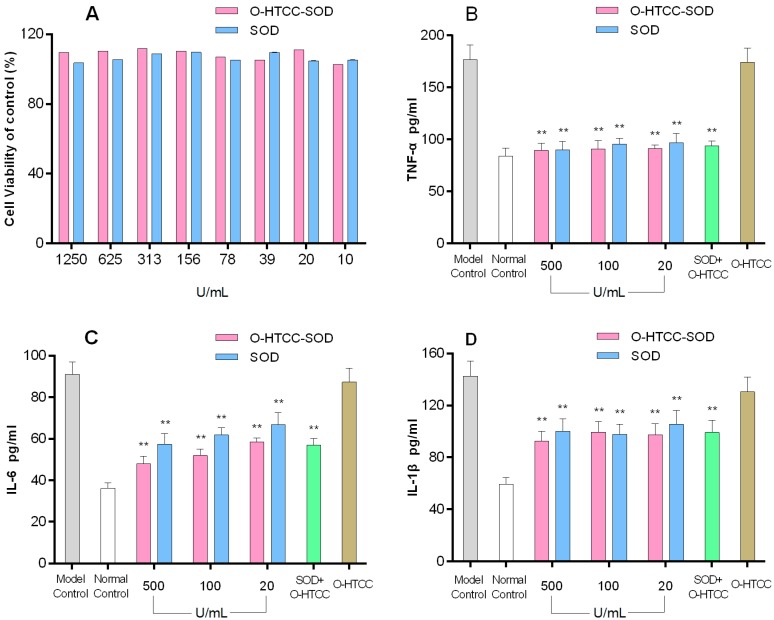
The effects of SOD and *O*-HTCC-SOD on: cell viability (**A**); and pro-inflammatory cytokine production (**B**–**D**) in LPS-stimulated murine peritoneal macrophages. Cell viability was measured using MTT (3-(4,5-dimethylthiazolyl-2)-2,5-diphenyl tetrazolium bromide) assay. ELISA was used to measure cytokines (TNF-α, IL-6 and IL-1β) in peritoneal macrophages after incubation with *O*-HTCC-SOD or SOD in presence of LPS. Model control and normal control refer to lipopolysaccharides (LPS)-treated or, non-treated cells respectively. Each determination was performed in quadruplicate. Data are presented as mean ± SD (*n* = 4). ** *p* < 0.01 compared with model control group.

Macrophages are major inflammatory and immune effectors cells, closely related to phagocytic cells, which cooperate during the onset, progression and resolution of inflammation [21]. In IBD and experimental colitis, disruption of the balance of the intracellular reduction–oxidation state leads to recruitment of monocytes in blood to the mucosa and differentiate into activated macrophages that produce pro-inflammatory cytokines, such as TNF-α, IL-1β, and IL-6 in the colonic mucosa or serum [22,23,24]. Therefore, it is vital to estimate the anti-inflammatory effect of *O*-HTCC-SOD on activated macrophage. To define the experimental dose range of *O*-HTCC-SOD for in vitro use, its effect on cell viability was assessed by MTT assay. As shown in Figure 3A, *O*-HTCC-SOD, as well as SOD, did not exert significant cytotoxic effects on peritoneal macrophage at a concentration ranging from 10 to 1250 U/mL. Thus the anti-inflammatory activity of *O*-HTCC-SOD (20, 100 and 500 U/mL) was confirmed in LPS-stimulated peritoneal macrophages in vitro. TNF-α, IL-1 β and IL-6 production was markedly increased in the culture supernatant of peritoneal macrophages after LPS stimulation, whereas the levels of these pro-inflammatory cytokines (Figure 3B–D) were significantly reduced by *O*-HTCC-SOD (*p* < 0.01). Besides, SOD and its mixture with *O*-HTCC had similar inhibitory activities on various cytokines (*p* < 0.01). Briefly, *O*-HTCC-SOD significantly inhibited LPS-induced inflammation response in macrophages by suppressing production of TNF-α, IL-6, and IL-1β, which attribute to superoxide anion removal by SOD enzyme. 

### 2.3. O-HTCC-SOD Inhibited Intracellular Reactive Oxygen Species (ROS) Production in LPS-Stimulated Macrophages

**Figure 4:**
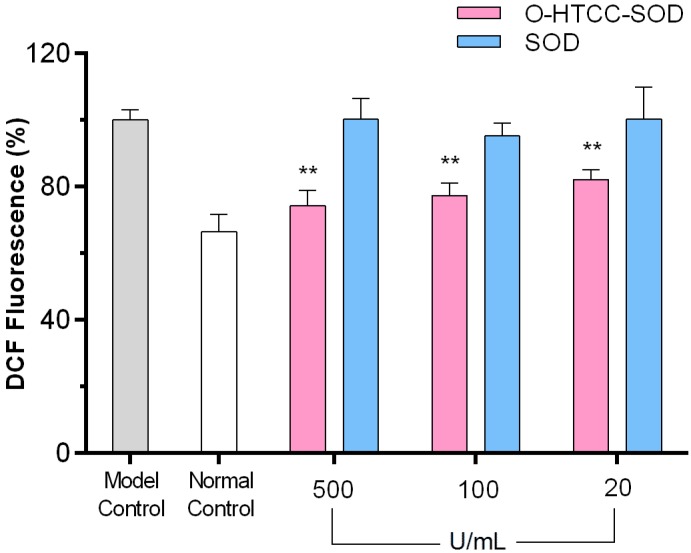
*O*-HTCC-SOD inhibited LPS-induced intracellular ROS production in murine peritoneal macrophages. Cells were pretreated with *O*-HTCC-SOD or SOD for 18 h, then stimulated with LPS (20 μg/mL) for additional 6 h. The intracellular ROS levels were determined by DCFH-DA method and expressed as percentage of fluorescence intensity at an excitation and emission wavelength of 485 and 535 nm compared to model control. The determination was performed in quadruplicate. Data are presented as mean ± SD (*n* = 4). ** *p* < 0.01 compared with model control group.

Oxygen free radicals are suggested to be signaling messengers in LPS-mediated inflammatory response [25,26,27]. Herein, the effect of *O*-HTCC-SOD on LPS-induced intracellular ROS production were estimated by using the fluorescent probe DCFH-DA, which can be oxidized to the highly fluorescent compound DCF. Our study demonstrated that, as illustrated in Figure 4, the peritoneal macrophages that were pre-treated with *O*-HTCC-SOD followed by exposure to LPS exhibited a remarkable decrease in intracellular ROS levels (*p* < 0.01). However, we did not observe a similar phenomenon for macrophages in any other group pre-treated with native SOD alone (*p* > 0.05). Presumably, the highly cationic *O*-HTCC incorporation endowed SOD with a strong positive charge, which was favorable to cell attachment and subsequent delivery of the enzyme into the cells where ROS could be removed. Poor membrane permeability of native SOD led to its weak capability to eliminate the intracellular ROS.

### 2.4. O-HTCC-SOD Attenuated the Severity of Dextran Sodium Sulfate (DSS)-Induced Colitis

**Figure 5:**
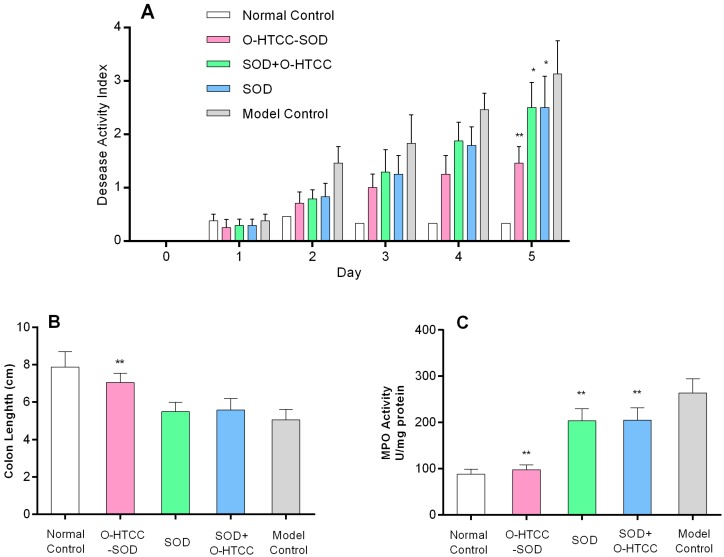
*O*-HTCC-SOD attenuated: DSS-induced experimental colitis severity (**A**,**B**); and colonic neutrophil infiltration (**C**). Mice in normal control had free access to drinking water without DSS. Other mice were treated with 5% DSS and intravenously administered for five days with normal saline (model control), SOD (3.0 kU/kg), SOD + *O*-HTCC (3.0 kU/kg + 0.1 mg/kg), or *O*-HTCC-SOD conjugate (3.0 kU/kg). DAI was measured daily (**A**). The length of the colon; and (**B**) colonic myeloperoxidase (MPO) activity (**C**) were determined at the end of the experimental period. Data are presented as mean ± SD (*n* = 8). * *p* < 0.05 or ** *p* < 0.01 compared with the model group.

It has been proven that DSS-induced colitis model is well-characterized by increased epithelial injury [18,28], and has many similarities with the clinical manifestations of colitis in human IBDs [29]. BALB/c mice fed 5% DSS in drinking water were monitored daily for clinical symptoms for five days. In mice exposed to DSS alone, a continuous increase in DAI score, clinically describing the severity of colitis, was observed from day 2 onwards (Figure 5A), indicating the stable occurrence of colitis in mice. The mice that were just given water were used as control. Intravenous administration of *O*-HTCC-SOD at 3 kU/kg significantly decreased the DAI score compared to DSS model mice (*p* < 0.01). Treatment groups of both the native SOD at 3 kU/kg and its mixture with *O*-HTCC (0.1 mg/kg) also showed statistically lower DAI scores than the DSS group (*p* < 0.05), but their effects were much lower than that of the *O*-HTCC-SOD conjugate. In addition, acute colitis was associated with a significant shortening of the colon. As illustrated in Figure 5B, the colon length of DSS-treated mice was reduced by 35.9% compared to that of healthy control mice. Treatment once daily with *O*-HTCC-SOD over days via intravenous administration significantly prevented against shortening of colon length (*p* < 0.01) induced by oral DSS. There was an inhibition trend towards mouse colon reduction for native SOD and its mixture with *O*-HTCC, nevertheless, protection did not reach significance compared to DSS-treated mice (*p* > 0.05). The results indicated that modification of SOD with *O*-HTCC significantly improved its capability to ameliorate the severity of DSS-induced intestinal damage as indicated by DAI score and colon length measurement.

### 2.5. O-HTCC-SOD Reduced Neutrophil Infiltration into the Colon of DSS-Treated Mice

It is scientifically proved that the development of acute inflammation was correlated with the activity of myeloperoxidase (MPO), an enzyme present in neutrophils and in much smaller quantities in monocytes and macrophages. MPO activity is considered as a biomarker of neutrophil infiltration in the acute inflammation [30]. Thus, colon inflammation was quantitatively assayed by assessment of MPO activity (Figure 5C). As expected, mouse colonic MPO activity was significantly increased (~3-fold) in the DSS control group compared with the normal control group (*p* < 0.01). In the *O*-HTCC-SOD group, however, the MPO activity was remarkably decreased compared with the DSS control group (*p* < 0.01) and was almost equivalent to the healthy control group. In contrast, a slight decrease in level of MPO activity was present in either native SOD group or its mixture with *O*-HTCC group compared with the DSS control group (*p* < 0.05). Therefore, *O*-HTCC-SOD therapy markedly reduced neutrophil accumulation within the colon of DSS-treated mice, and the efficacy was much better than the native SOD.

### 2.6. O-HTCC-SOD Diminished Colonic Histopathological Changes

**Figure 6:**
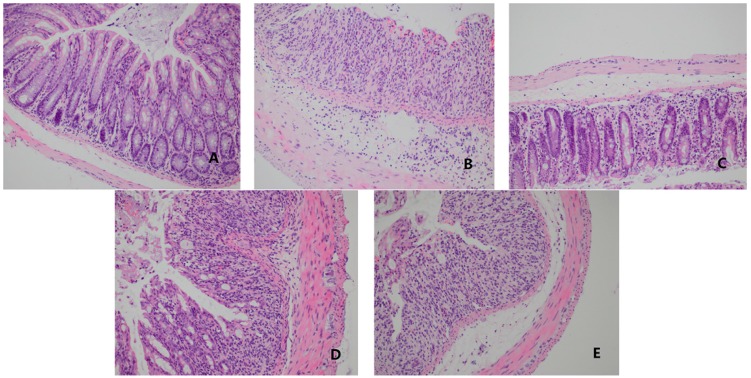
*O*-HTCC-SOD diminished colonic histopathological changes. DSS-induced colonic inflammation and mucosal damage were assessed by hematoxylin–eosin staining (H&E staining, 200×). Mice in normal control (**A**) had free access to drinking water without DSS. Other mice were treated with 5% DSS and intravenously administered for five days with normal saline: model control group (**B**); *O*-HTCC-SOD (3 kU/kg) (**C**); native SOD (3 kU/kg) (**D**); or SOD + *O*-HTCC (3 kU/kg + 0.1 mg/kg) (**E**).

DSS-induced colonic inflammation and mucosal damage were assessed by detecting the morphological alterations of the mouse colon after H&E staining. Representative stained sections were shown in Figure 6. Tissue sections from representative areas of colon in normal control group showed normal colonic histology with an intact surface epithelium, well-defined gland lengths and no neutrophil infiltration in the mucosa (Figure 6A). Mice fed DSS in drinking water showed typical characteristics of abnormal colon structure, as indicated by loss of epithelial and goblet cells, crypt lesions and massive area with prominent inflammatory cell infiltration in the mucosa and submucosa (Figure 6B). Intravenous injection of *O*-HTCC-SOD at 3 kU/kg obviously restored the architecture of the mouse colon epithelium with a marked decrease in inflammatory cell infiltration compared with the DSS group (Figure 6C). By contrast, there was marginally protective effect against the structural damage of colonic tissue caused by DSS in native SOD group (Figure 6D) or SOD + *O*-HTCC mixture group (Figure 6E).

### 2.7. Dose–Response Profile of O-HTCC-SOD

**Figure 7:**
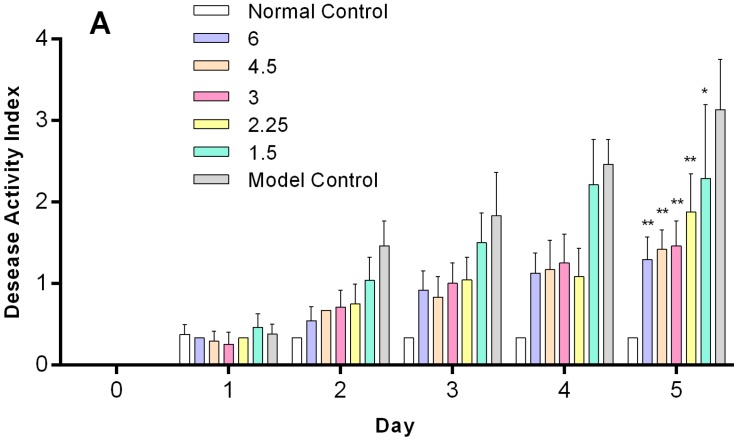

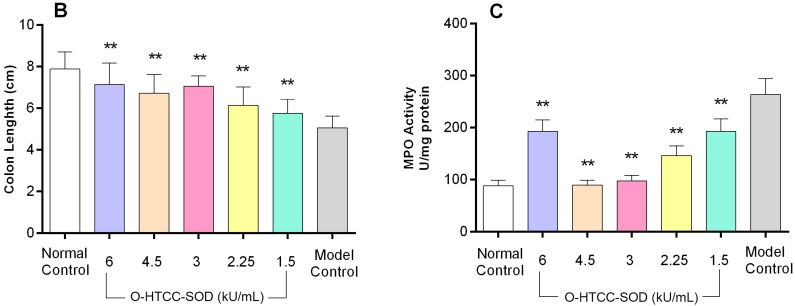
Dose–response profile of *O*-HTCC-SOD on DSS-induced colitis. Mice in normal control had free access to drinking water without DSS. Other mice were treated with 5% DSS and intravenously administered with normal saline (model control) or *O*-HTCC-SOD (1.5, 2.25, 3.0, 4.5, or 6 kU/kg) for five days. DAI was measured daily (**A**). The length of the colon (**B**); and colonic MPO activity (**C**) were determined at the end of the experimental period. Data are presented as mean ± SD (*n* = 8). * *p* < 0.05 or ** *p* < 0.01 compared with the model group.

To determine the preliminary dose–response profile of *O*-HTCC-SOD conjugates, we compared the therapeutic effects of conjugates at different doses (1.5–6.0 kU/kg) on the development of colitis induced by 5% DSS administration. *O*-HTCC-SOD were intravenously administered once daily for five days. As shown in Figure 7A, *O*-HTCC-SOD produced beneficial effect on DAI, but showed no obvious dose-dependence at 1.5 to 6.0 kU/kg. In the case of colon shortening (Figure 7B), the maximal effect was observed in response to 3.0 kU/kg *O*-HTCC-SOD, whereas higher doses (4.5–6.0 kU/kg) had no significant effect. Moreover, *O*-HTCC-SOD at 3.0 to 4.5 kU/kg was detected less level on colonic MPO activation, rather than lower doses (1.5–2.25 kU/kg) or 6 kU/kg (Figure 7C). In summary, we could draw a conclusion that *O*-HTCC-SOD at 3.0 kU/kg had the best protective effect against DSS-induced colitis in mice.

## 3. Materials and Methods

### 3.1. Materials

Cu/Zn superoxide dismutase (SOD) was obtained from Chengdu Kemaipu Biotechnology Co., Ltd. (Chengdu, China). Chitosan (*M*w = ∼50 kDa, >90% degree of deacetylation) was supplied by Haidebei Marine Bioengineering Co., Ltd. (Jinan, China). Glycidyl trimethylammonium chloride (GTMAC), 1-ethyl-3-(3-dimethylaminopropyl) carbodiimide hydrochloride (EDC), *N*-hydroxy sulfosuccinimide sodium (NHS), 3-(4,5-dimethylthiazolyl-2)-2,5-diphenyl tetrazolium bromide (MTT), Lipopolysaccharides (LPS, from *E. coli* O55:B5) were purchased from Sigma-Aldrich (St. Louis, MO, USA). DEAE Sepharose Fast Flow gel was from GE healthcare (Uppsala, Sweden). Dulbecco’s Modified Eagle’s medium (DMEM), and fetal bovineserum (FBS) were from HyClone, (Logan, UT, USA). TNF-α, IL-6 and IL-1β ELISA kits were from Bio-Swamp (Wuhan, China). Dextran Sulfate Sodium salt (DSS, *M*_W_ = 36–50 kDa) was from MP Biomedicals (Irvine, CA, USA). ROS Assay Kit was from Beyotime (Nanjing, China). All other chemicals were of general reagent grade (unless stated).

### 3.2. Experimental Animals

Male BALB/c mice (6–8 weeks old) were purchased from Beijing HFK Bioscience Co., Ltd. (Beijing, China; Quality certificate Number: 11401300051370). The mice were maintained under regular laboratory conditions (i.e., room temperature and natural light–dark cycle) for 1 week before study, with free access to standard rodent chow and water. All animal protocols used in this study were approved (ethic approval No. LL-201402083; 12 May 2014) and strictly conducted in compliance with the institutional guidelines for the Animal Care and Use Committee of Shandong University (Jinan, China).

### 3.3. Preparation of O-HTCC-SOD Conjugate

The *O*-quaternized chitosan derivative, *O*-HTCC, was synthesized using an established method previously [31] with slight modifications. Briefly, 100 mL of 3% (*w*/*v*) chitosan solution (~18.5 mmol of glucosamine residues) in 10% (*v*/*v*) acetic acid was reacted with 15 mL of benzaldehyde (8.0 equiv.) under stirring for 1 h at room temperature, concentrated in vacuo at 60 °C for 20 h, neutralized by 1.0 M NaOH, followed by filtration, repeated washing with methanol and vacuum drying to give amino-protected N-benzylidene chitosan. To the chitosan intermediates in 50 mL isopropyl alcohol were added GTMAC (7.96 mL, 59.4 mmol, 3.0 equiv.) by dropwise, stirred under reflux at 70 °C for 16 h. The filtered precipitate was washed with methanol and acetone alternately, dried in vacuo, and then dispersed in 50 mL of 0.25 M HCl ethanol solution. The mixture was stirred at room temperature for 24 h, followed by concentration on a rotary evaporator to remove the ethanol. The solution was diluted with 15 mL H_2_O, precipitated overnight in acetone. The pellets were dissolved in some distilled water, concentrated by ultrafiltration using a LabScale TFF System (Merck Millipore, Billerica, MA, USA) equipped with a 10 kDa molecular weight cut-off (MWCO) membrane to remove impurities, and further lyophilized to obtain *O*-(2-hydroxyl) propyl-3-trimethyl ammonium chitosan chloride (*O*-HTCC, 2.76 g). The amino protective and deprotective reactions were monitored on a Nicolet Nexus 470 FT-IR spectrometer (Thermo Fisher Scientific, Waltham, MA, USA). The final products were characterized using ^1^H NMR to confirm the identity, followed by determination of the percentage of free amino groups in *O*-HTCC using a ninhydrin colorimetric method [32].

Cu/Zn superoxide dismutase (SOD) was conjugated with *O*-HTCC to give a novel polymer–enzyme conjugate, *O*-HTCC-SOD, via a carbodiimide-mediated reaction established by our laboratory previously [17]. Briefly, 1.0 mg/mL of SOD in 25 mM phosphate buffer (pH 6.0) was incubated with >10-fold molar excess of EDC and NHS, with slow stirring for 30 min at room temperature, and then terminated by 2-mercaptoethanol at a final concentration of 20 mM. Next, 1.0 mg/mL of *O*-HTCC in 25 mM phosphate buffer (pH 6.0) was slowly added into the above activated SOD with slow stirring. The reaction lasted in dark for 8 h at room temperature. The reaction mixture was purified on a DEAE Sepharose Fast Flow chromatography column (2.6 × 40 cm) on an ÄKTA avant chromatography system equipped with a UV detector (GE healthcare, Chicago, IL, USA), eluting at a flow rate of 1.5 mL/min successively with a gradient of 5 → 100 mM phosphate buffer (pH 8.0). The fractions of interest were pooled, desalted by ultrafiltration and lyophilized to get the *O*-HTCC-SOD conjugate as white fluffy floc. The resulting *O*-HTCC-SOD was further identified to be polydisperse with higher molecular weights than SOD. These data are same to our previous results [17].

### 3.4. Enzymatic Activity Assay

SOD activity was performed by pyrogallol autoxidation method as described before [17]. Briefly, a sample in 50 mM Tris-HCl buffer (pH 8.2) containing 1 mM Na_2_EDTA was thoroughly mixed with an appropriate volume of 50 mM pyrogallol in 10 mM HCl. The final volume was adjusted to 4.5 mL using the Tri-HCl buffer. The absorbance at 325 nm (A_325_) of the mixtures was measured every 48 s for 4 min at 37 °C respectively. The pyrogallol autoxidation rate was expressed as the change of A_325_ per minute. One unit of SOD activity was the amount of the enzyme that inhibited the autoxidation rate by 50% (IC_50_) per minute and SOD specific activity was expressed as units per mg protein per min. In this study, the protein content was determined using the BCA assay compared to a bovine SOD standard. The residual activity of *O*-HTCCSOD was expressed as the percentage of unit number of unmodified SOD.

### 3.5. Stability Studies

The native or *O*-HTCC conjugated SOD at equivalent enzyme activity was dissolved separately in 50 mM phosphate buffers with varying pH levels (2–13) and allowed to incubate at 37 °C for 30 min. The relative activity was expressed as the percentage of the original activities. Similarly, the thermal stability was investigated by determining respectively the percentage residual activity of SOD and conjugates in 50 mM phosphate buffer (pH 7.4) that were incubated for 30 min at different temperatures (25–80 °C). To assess the stability to proteolytic degradation, pepsin or trypsin was added to the conjugate solution respectively, and incubated at 37 °C for 30 min. Aliquots were taken at scheduled times and assayed for relative enzymatic activity (%) compared to initial activity. Finally, we monitored 30-day activity changes of native SOD and *O*-HTCC-SOD at room temperature to estimate their long-term stability for storage.

### 3.6. Preparation and Viability Assay of Mouse Peritoneal Macrophages

Starch-elicited peritoneal macrophages were prepared from BALB/c mice using previousmethod with slight modifications [33]. Briefly, male BALB/c mice were each injected intraperitoneally with 2.5 mL of sterile 3% starch (*w*/*v*) in distilled water to cause an inflammatory response and to recruit the macrophages. Three days later, mice were sacrificed by cervical dislocation and the peritoneal exudate cells were isolated by centrifugation at 1000 rpm for 5 min. The cells were suspended in RPMI-1640 medium supplemented with 10% fetal bovine serum and maintained at 37 °C in a humidified incubator containing 95% air and 5% CO_2_. In the following experiments, cells at the desired concentration were seeded in appropriate cell plates at 37 °C for 2 h to the bottom of the wells. Non-adherent cells were removed by extensive washing with RPMI-1640 medium. Virtually all of the adherent cells were macrophages, as previously described [34].

The macrophage viability was measured based on the mitochondrial-dependent MTT reduction assay originally described by Mosmann [35] with slight modifications. Briefly, the adhering macrophages in a 96-well plate at 1 × 10^5^ cells per well were treated with different concentrations of unmodified SOD or *O*-HTCC-SOD at 37 °C for 24 h, and then incubated with MTT for additional 4 h. After solubilization in dimethyl sulfoxide (DMSO), the extent of reduction of MTT to formazan was quantitated by measuring the absorbance at 490 nm in a microplate reader (Bio-Rad, Hercules, CA, USA). Cell viability was expressed as percentage (%) of absorbance in experimental groups compared to untreated cells. Data represented average ± SD. The experiment was carried out in triplicate.

### 3.7. LPS-Induced Cytokine Production in Macrophages

Murine peritoneal macrophages at 1 × 10^5^ cells/well were seeded in 96-well plates for 2 h, and treated for 24 h with SOD or *O*-HTCC-SOD at different concentrations (20, 100, 500 U/mL), SOD (500 U/mL) + *O*-HTCC (500 U/mL + 20.0 μg/mL), *O*-HTCC (20.0 μg/mL) in presence of 1.0 μg/mL of LPS (from *Escherichia coli* serotype O55: B5). Cell culture media were collected, and the levels of secreted TNF-α, IL-6, and IL-1β in supernatants were measured using ELISA kits according to the manufacturer’s instructions. Each determination was performed in quadruplicate.

### 3.8. LPS-Induced ROS Production in Macrophages

The effect of *O*-HTCC-SOD on intracellular ROS production in LPS-activated macrophage was determined by using the fluorescent probe 2′,7′-dichlorofluorescein diacetate (DCFH-DA), which freely permeates cells, and upon incorporation, is converted, by oxidation, to the fluorescent 2,7-dichlorofluorescein (DCF) [36]. The peritoneal macrophages (5 × 10^4^ cells/well) seeded in 96-well culture plates were pre-treated for 18 h with SOD and *O*-HTCC-SOD at a final concentration of 20, 100, 500 U/mL, respectively. After removal of the culture media, cells were washed with PBS for three times, and then were exposed to LPS (20 μg/mL) in fresh medium with for another 6 h. Macrophages were harvested, washed twice with cold PBS and incubated in dark with 10 μM DCFH-DA at 37 °C for 30 min, followed by washing with RPMI-1640 medium three times. DCF fluorescence within the cells was measured with a fluorescence microplate reader (EnSpire, PerkinElmer, Waltham, MA, USA) at an excitation and emission wavelength of 485 nm and 535 nm, respectively. The intracellular ROS levels were expressed as percentage of DCF fluorescence compared to model control.

### 3.9. Models of Experimental Colitis and Treatment

Seventy-two BALB/c mice were randomly divided into nine groups, each group consisting of eight animals. One group was assigned to the normal control group and had free access to normal drinking water without DSS during the whole course of this experiment. The remaining eight groups were used to induce acute colitis models by administration of 5% (*w*/*v*) DSS (36–50 kDa, MP Biomedicals, Irvine, CA, USA) in drinking water ad libitum for 5 consecutive days as described previously [28]. Mice in each experiment group were treated by intravenous administration into tail vein for five days as follows: DSS model group (normal saline), SOD (3.0 kU/kg), SOD + *O*-HTCC (3.0 kU/kg + 0.1 mg/kg), and *O*-HTCC-SOD conjugate (1.5, 2.25, 3.0, 4.5, 6 kU/kg) groups. The first administration of these drugs was done just before the start of DSS administration. The injection volume was 0.2 mL.

### 3.10. Evaluation of Colitis Progression

The colitis progression and severity was assessed using the disease activity index (DAI) as described by Cooper et al. [37] with a slight modification. During the duration of the experiment, mice body weight, stool consistency, and gross bleeding were recorded daily. The DAI was determined as one-third of scores of body weight loss compared to initial weight, stool consistency, and gross bleeding. Each score was determined as follows: change in body weight loss (0: none, 1: 1–5%, 2: 6–10%, 3: 11–15%, 4: >15%), stool blood (0: negative, 1: +, 2: ++, 3: +++, 4: ++++) and stool consistency (0: normal, 1 and 2: loose stool, 3and 4: diarrhea). DAI can be scored daily during the duration of the DSS treatment. Upon completion of the experiment, all animals were sacrificed via cervical dislocation immediately. The entire colon, from the caecum to the anus, was removed post-mortem, and the colon length was measured as a marker of inflammation [38]. Then, the colon was cut into sections and stored at −80 °C until use.

### 3.11. Detection of MPO Activity in Colon Tissues

The colonic myeloperoxidase (MPO) activity was determined to assess neutrophil infiltration into the colon [39]. Briefly, a given amount of the thawed colon segments was homogenized on ice in appropriate volume of phosphate buffer, followed by quantitative measurement of MPO activity of the homogenate by using tetramethylbenzidine (TMB) as substrate according to the instruction of a MPO assay kit (Nanjing Jiancheng Bioengineering Institute, Nanjing, China). The absorbance was measured at 460 nm using a Cary 60 UV-Vis Spectrophotometer (Agilent, Santa Clara, CA, USA). Myeloperoxidase activity is expressed as the amount of enzyme required to convert 1 μmol of hydrogen peroxide to water, expressed per gram of wet weight of tissue.

### 3.12. Histopathological Analysis

The distal colonic segments were fixed in 10% buffered formalin solution overnight at 4 °C, dehydrated in serial alcohol solutions, and embedded in paraffin. Paraffin sections were cut into 5-μm thick slices, stained with Hematoxylin and Eosin (H&E), and finally visualized under a light microscope (Nikon, Tokyo, Japan; with 200× magnification) to evaluate damage to the intestinal mucosa was evaluated.

### 3.13. Statistics

One-way analysis of variance (ANOVA) or unpaired Student’s *t*-test was used for statistical comparisons among the groups using SigmaPlot 13.0 analysis software (San Jose, CA, USA). The data were expressed as means ± standard deviation (SD). Differences with *p* < 0.05 were considered to be statistically significant, and *p* < 0.01 extremely significant.

## 4. Conclusions

SOD has attracted considerable attention as a candidate for treatment of various severe diseases that are related with ROS. Given the clinical limitations of SOD, a novel polymer–enzyme conjugate (*O*-HTCC-SOD) has been synthesized at our lab through covalent coupling of SOD to *O*-(2-hydroxyl) propyl-3-trimethyl ammonium chitosan chloride (*O*-HTCC). The *O*-HTCC-SOD demonstrated its versatile advantage over native SOD, including superior cellular uptake, longer half-life and better bioavailability, as well as enhanced protective effect against ROS-induced oxidative damage in vivo. To further explorer therapeutic potential, the present paper presented the stability of *O*-HTCC-SOD towards a variety of environmentally relevant stressors. Our data demonstrated that *O*-HTCC-SOD had wider pH activity range, better thermal stability and excellent long-term stability for storage. Interestingly, *O*-HTCC-SOD has a unique reinstatement of activity exposure to pepsin degradation, which was related to the gradual degradation of *O*-HTCC coupled with SOD and was helpful for the enzyme to prolong half-life and enhance the bioavailability. In vitro studies exhibited that *O*-HTCC-SOD significantly down-regulated production of pro-inflammatory cytokines (TNF-α, IL-6, IL-1β) in LPS-stimulated mouse peritoneal macrophages, also reduced intracellular ROS levels due to superior membrane permeability to native SOD. Finally, a well-established DSS-induced colitis model was used to investigate the therapeutic effect of *O*-HTCC-SOD. *O*-HTCC-SOD significantly attenuated the severity of DSS-induced colitis in mice as observed by decreased disease activity index (DAI) and lower colon length, reduced neutrophil infiltration into the colon according to myeloperoxidase (MPO) activity assay, and diminished colonic histopathological damage, whereas native SOD failed to do so. Our data suggests that conjugation of *O*-quaternary chitosan derivative *O*-HTCC enhanced the therapeutic potential of SOD. This research has important implications for *O*-HTCC-SOD as a promising therapeutic option in the management of inflammatory bowel disease or other ROS-related diseases.
